# Rottlerin-induced autophagy leads to the apoptosis in breast cancer stem cells: molecular mechanisms

**DOI:** 10.1186/1476-4598-12-171

**Published:** 2013-12-23

**Authors:** Dhruv Kumar, Sharmila Shankar, Rakesh K Srivastava

**Affiliations:** 1 Department of Pharmacology, Toxicology and Therapeutics, and Internal Medicine, The University of Kansas Medical Center, 3901 Rainbow Boulevard, Kansas City, KS 66160, USA; 2 Department of Pathology and Laboratory Medicine, The University of Kansas Medical Center, 3901 Rainbow Boulevard, Kansas City, KS 66160, USA

**Keywords:** 3-methyladenine (*3-MA*), Autophagy, Bafilomycin (Baf), Beclin-1, Cycloheximide (CHX), LC3, AMPK, Atg12

## Abstract

**Background:**

Autophagy is an indispensable lysosomal self-digestion process involved in the degradation of aggregated proteins and damaged organelles. Autophagy is associated with the several pathological processes, including cancer. Cancer stem cells (CSCs) play significant roles in cancer initiation, progression and drug resistance. Recent studies have demonstrated the antitumor activities of plant-derived chemopreventive agent rottlerin (Rott). However, the molecular mechanism by which Rott induces autophagy in breast CSCs has not been investigated.

**Results:**

The objectives of this study were to examine the molecular mechanism by which Rott induces autophagy which leads to apoptosis in breast CSCs. Treatment of breast CSCs with Rott for 24 h resulted in a concentration dependent induction of autophagy, followed by apoptosis as measured by flow cytometry. Electron microscopy confirmed the presence of autophagosomes in Rott treated breast CSCs. Western blot analysis showed that Rott treatment increased the expression of LC3, Beclin-1 and Atg12 that are accumulated during autophagy. Prolonged exposure of breast CSCs to Rott caused apoptosis which was associated with the suppression of phosphorylated Akt and mTOR, upregulation of phosphorylated AMPK, and downregulation of anti-apoptosis Bcl-2, Bcl-X_L_, XIAP and cIAP-1. Knock-down of Atg7 or Beclin-1 by shRNA inhibited Rott-induced autophagy at 24 h. Our study also demonstrates that pre-treatment of breast CSCs with autophagosome inhibitors 3-methyladenine and Bafilomycin, as well as protein synthesis inhibitor cycloheximide inhibited Rott-induced autophagy and apoptosis. Rott induces autophagy via extensive cytoplasmic vacuolization in breast CSCs. Molecular docking results between C2-domain of protein kinase C-delta and Rott indicated that both hydrogen bonding and hydrophobic interactions contributed significantly for ligand binding with minimum binding affinity of ≈ 7.5 Kcal/mol. Although, autophagy inhibitors suppress the formation of cytoplasmic vacuolization and autophagy in breast CSCs, the potency of Rott to induce autophagy and apoptosis might be based on its capability to activate several pathways such as AMPK and proteasome inhibition.

**Conclusions:**

A better understanding of the relationship between autophagy and apoptosis would eventually allow us to discover novel drugs for the treatment of breast cancer by eliminating CSCs.

## Background

Autophagy is a highly conserved cellular process that is involved in several catabolic processes, cellular development [1], autoimmunity [2], degradation of long-lived proteins and organelles [3], and cell death [4]. It has also been involved in several other cellular mechanism which are directly or indirectly related to diseases like neurodegeneration, cardiovascular, aging and cancer [5]. Autophagy takes place at basal levels in most of the cell types but is also regulated developmentally and/or by environmental stimuli. Autophagy is upregulated when cells encounter environmental stressors such as nutrient starvation, pathogen infection and chemotherapeutic agents, [6-9] and the process is essential for the maintenance of cellular energy, and thereby, for cell survival in stress conditions [10,11]. Although autophagy is initiated as a protective response to stress, the constitutive activation of autophagy can lead to cell death by excessive self-degradation of essential cellular components [12].

Recently, it has been reported that the chemotherapeutic agents [13,14] induced the early stage of autophagy in cancer stem cells (CSCs) [15,16], and it is regulated by several ‘Atg’ (Autophagy-related) genes [17] and proteins which have been implicated in autophagosome formation [18]. Autophagosome nucleation requires a complex containing Atg6, whereas elongation of autophagosome involves Atg12 and Atg8 (LC3 in mammals) [19]. Atg7 is required to recruit other proteins to the autophagosomal membrane and to form the autophagic vacuole in a pathway [20,21]. All together, they form autophagic membrane; this membrane assembles around damaged organelles, proteins and cytoplasm. Later, the outer membrane of autophagosomes is fused by endosomes or lysosomes to form autolysosomes where lysosomal hydrolases degrade the cytoplasm derived contents of autophagosome together with its inner membrane and presented to citric acid cycle for energy generation [22]. Moreover, an important autophagy-regulatory gene such as Beclin-1 functions as a haplo-insufficient tumor suppressor gene [23], further emphasizing the clinical importance of autophagic cell death and apoptosis.

Despite of these advances, the relationship between autophagy and apoptosis in CSCs is still not well understood. CSCs may be responsible for tumor onset, self-renewal/maintenance, mutation accumulation, and metastasis [24]. In CSCs, autophagy plays an important role in the regulation of drug resistance, self-renewal, differentiation, and tumorigenic potential [25,26], suggesting autophagy could be a promising therapeutic target in a subset of cancers. In some circumstances, both autophagy and apoptosis have been observed in the same cells, [27-30] and they may be interconnected by some signaling pathway [17,31]. The Akt/mTOR and AMPK signaling pathway is a key regulator of physiological cell processes which include proliferation, differentiation, apoptosis, motility, metabolism, and autophagy. Several anti-apoptotic signals such as the Akt/mTOR signaling pathway, and Bcl-2 suppress autophagy [17,32] and concurring-apoptotic signals such as the AMPK signaling pathway, and Bax activate autophagy [33]. Conversely, autophagy may inhibit apoptosis, [34,35] and the inhibition of autophagy can activate apoptosis [28,31,36].

Autophagy also plays an essential role in the maintenance of cellular energy and for cell survival in stress conditions [10,11]. Endoplasmic Reticulum (ER) stress and activation of AMPK are among the major regulators of autophagy [37], which are involved in biosynthesis, protein folding and modification of various soluble and insoluble proteins [38]. The ER-resident proteins, PERK and IRE1, and increased cytosolic calcium have been implicated as mediators of ER stress induced autophagy in mammalian cells [39]. These mediators activate autophagy by upregulating Atg12 and LC3 conversion [40]. ER stress also leads to release of calcium from ER to cytosol, which in turn can activate various kinases that are involved in autophagy signaling [41,42]. Calcium mediated autophagy is regulated by AMP activated protein kinase (AMPK), which senses cellular energy status to maintain homeostasis. It is usually activated when ATP levels are depleted in the cells. Increase in the cytosolic calcium leads to the activation of Ca2+/calmodulin activating kinase kinase β (CAMKKβ) which further activates AMPK [43]. In addition, both AMPK and mTOR regulate autophagy through coordinated phoshphorylation of Ulk1 [44,45]. Thus activating autophagy may abolish the resistance of CSCs to chemotherapy and could lead to the development of novel therapeutic approaches for the treatment of various cancers.

Rott has been used as a protein kinase C-delta signaling pathway inhibitor [46]. It inhibits cell proliferation and induces apoptosis through mitochondrial membrane depolarization. Recently, in several human cancer cells, Rott has been shown to induce a starvation response, which is a key regulator of autophagy causing its induction [47]. We have recently reported the existence and role of human pancreatic CSCs in autophagy leading to apoptosis induced by Rott [26,48]. Since breast cancer contains rare breast CSCs, we sought to examine the molecular mechanism by which Rott induces autophagy in breast CSCs. Breast cancer is one of the leading gynecological cancers with high mortality rates. It is usually detected in late stages with poor prognosis. Here we report that Rott-induced early autophagy is mainly dependent on the induction of autophagosomes, conversion of LC3-I to LC3-II, expression of Atg12 and Beclin-1 and inhibition of Bcl-2, Bcl-xL, XIAP and cIAP-1. Eventually, Rott induced apoptosis through the inhibition of Akt/mTOR pathway, and activation of caspases and AMPK pathways. Moreover, expression of Atg12 and Beclin-1 enhanced apoptosis-inducing potential of Rott. These findings strongly suggest that Rott-induced autophagy may play some important role in induction of apoptosis. For the first time we report that Rott activates autophagy by inducing the phosphorylation of AMPK. We show a novel function of Rott that is involved in inducing early autophagy and late apoptosis in breast CSCs.

## Results

### Rottlerin induced cytoplasmic vacuolation and cell death in breast CSCs

**Figure 1:**
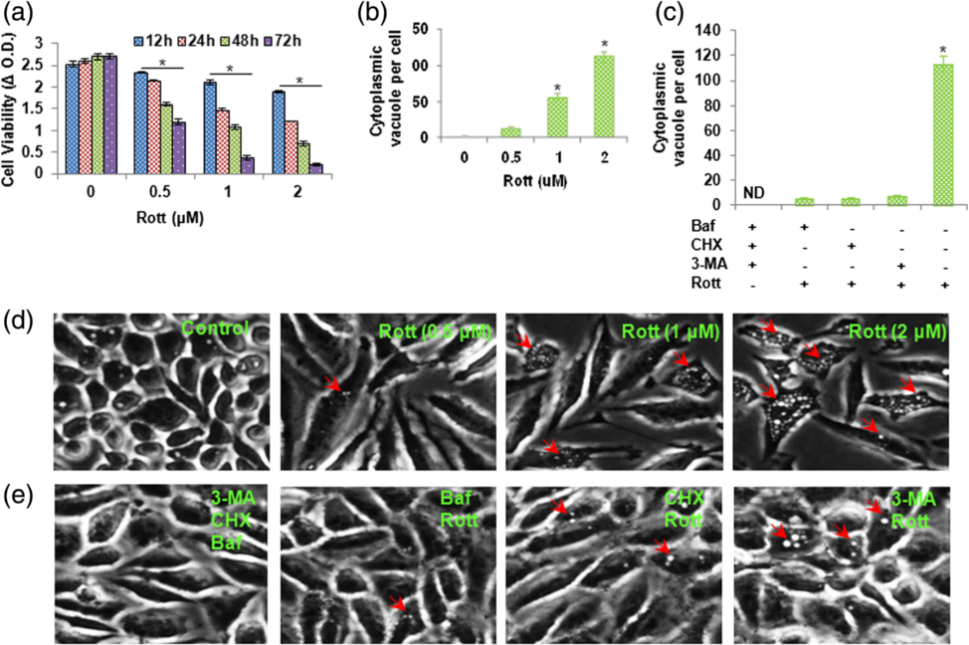
**Rottlerin inhibited cell viability, and induced apoptosis and cytoplasmic vacuolation in breast CSCs. (a)** Cells were grown in complete stem cell culture medium and treated with Rott (0, 0.5, 1 and 2 μM) for different time points. Cell viability was measured by XTT assay. Blue color (12 h), red (24 h), green (48 h), and violet (72 h). **(b)** Breast CSCs were treated with Rott (0, 0.5, 1 and 2 μM) for 48 h, and the autophagic vacuoles were counted under phase contrast microscope. **(c)** Breast CSCs were co-treated with Rott (2 μM) and Baf (10 nM), 3-MA (10 mM) or CHX (10 μg/ml) for 48 h, and the autophagic vacuoles were counted under phase contrast microscope. **(d)** Breast CSCs were cultured in complete stem cell culture medium and treated with the different concentrations of Rott (0, 0.5, 1 and 2 μM) for 48 h. Representative images were obtained by phase contrast microscopy. Magnification, 20X. Red arrows indicate cytoplasmic vacuoles developed by the effect of different concentration of Rott (0, 0.5, 1 and 2 μM). **(e)** Breast CSCs were cultured in complete stem cell culture medium and co-treated with Rott (2 μM) and Baf (10 nM), 3-MA (10 mM) or CHX (10 μg/ml) for 48 h. Representative images were obtained by phase contrast microscopy. Magnification, 20X. Red arrows indicate cytoplasmic vacuoles. Data are reported as the mean ± standard error (SE) of percentage of cells. *n* = 5, *P < 0 · 05 when compared with Rott treated in an identical manner.

To examine whether Rott induces cytotoxic effect on breast CSCs, we treated breast CSCs with different concentrations of Rott (0, 0.5, 1 and 2 μM) for various time points (12, 24, 48 and 72 h). Rott inhibited cell viability in a time- and dose-dependent manner (Figure 1a). While the treatment with 0.5 μM Rott had little effect on cell viability, treatments with 1 or 2 μM Rott for 48 and 72 h significantly inhibited cell viability. We next measured cytoplasmic vacuolation induced by Rott (Figure 1b and d). Rott induced autophagy in breast CSCs by forming cytoplasmic vacuolation in a dose-dependent manner. 1 μM and 2 μM Rott induced more cytoplasmic vacuolatyion in breast CSCs compared to 0.5 μM (Figure 1b and d). Whereas, co-treated breast CSCs with Rott and Baf, 3-MA or CHX inhibited cytoplasmic vacuolation (Figure 1c and e). Moreover, the breast CSCs treated with Rott showed morphological features of cytoplasmic vacuole accumulation. Rott increased more numbers of vacuole formation in the cytoplasm of breast CSCs (Figure 1d).

### Rottlerin induced early stage autophagy in breast CSCs

**Figure 2:**
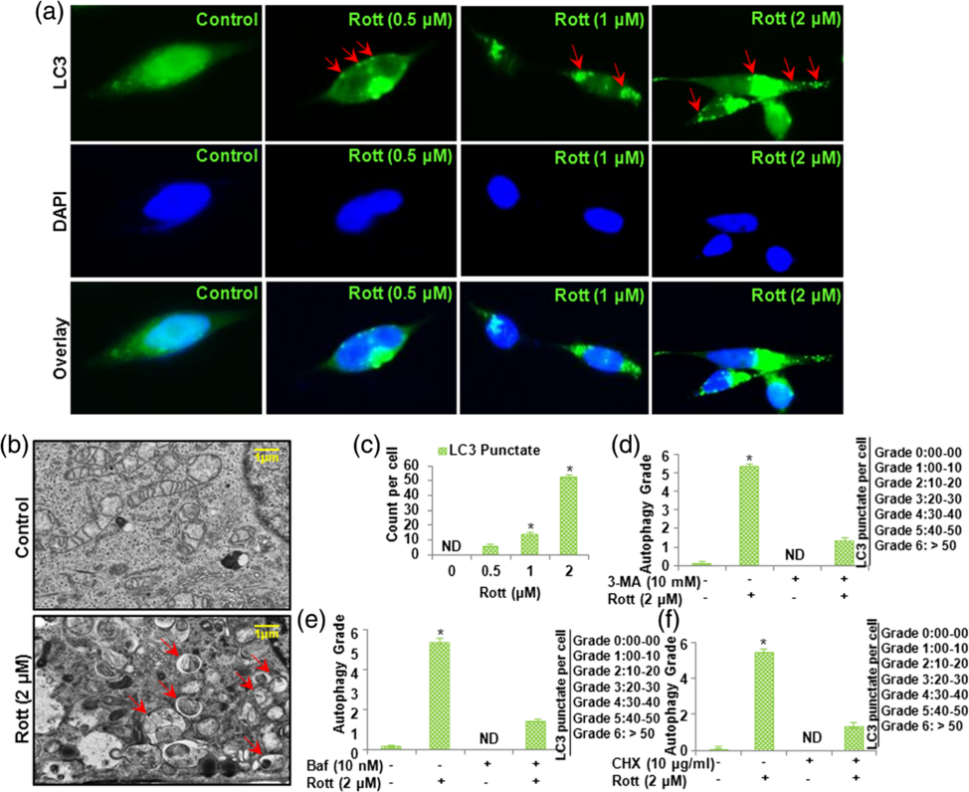
**Autophagic cell-death by rottlerin in breast CSCs. (a)** Redistribution of pEGFP-LC3. Breast CSCs were stably transfected with a pEGFP-LC3 fusion construct and cultured in complete stem cell culture medium, and treated with Rott (0, 0.5, 1 and 2 μM) for 48 h. Cells were visualized under a fluorescence microscope to examine the expression of LC3-II. LC3 expression increases by increasing Rott concentration in breast CSCs. **(b)** Electron microscopy shows the ultrastructure of breast CSCs treated with different concentrations of Rott (0 and 2 μM) in complete stem cell culture medium for 48 h. Arrows indicate autophagosomes including residual digested material (red arrow). **(c)** Punctate dot quantification in pEGFP-LC3-positive breast CSCs treated with Rott (0, 0.5, 1 and 2 μM) for 48 h. Quantification of puncatate dot per cell based on number of punctuate dot in pEGFP-LC3-positive cells. Quantification represents at least 100 cells counted and scored per treatment. **(d, e and f)** Punctate dot quantification in pEGFP-LC3-positive breast CSCs co-treated with Rott (2 μM) and Baf (10 nM), 3-MA (10 mM) or CHX (10 μg/ml) for 48 h. Quantification of puncatate dot per cell based on number of punctuate dot in pEGFP-LC3-positive cells. Quantification represents at least 100 cells counted and scored per treatment. Data are reported as the mean ± standard error (SE) of percentage of cells. *n* = 5, *P < 0 · 05 when compared with Rott treated in an identical manner.

LC3 is a hallmark of autophagy and the conversion of LC3-I to LC3-II via proteolytic cleavage and lipidation shows autophagy induction. Therefore, to study whether Rott induced autophagy in breast CSCs, the formation of LC3 punctate dots and conversion of LC3-I to LC3-II were examined by different molecular technique. This modification of LC3 is essential for the formation of autophagosomes and for the completion of macroautophagy. To confirm whether LC3 (autophagosomes) is redistributed after Rott treatment, we observed the induction of LC3 punctate dots in LC3 transfected breast CSCs with the exposure of different concentration of Rott (Figure 2a). Cells were cultured in complete stem cell culture medium, treated with or without Rott and subjected to immunofluorescence for visualization of pEGFP-LC3 transfected cells. Our results indicated that Rott induced autophagy in a dose dependent manner (Figure 2a). To examine whether cell vacuolation induced by Rott is related to autophagy, breast CSCs were treated with Rott (0, 0.5, 1 and 2 μM) for 48 h and the ultrastructure of cells were analyzed by electron microscopy. Numerous autophagic vacuoles containing lamellar structures or residual digested material and empty vacuoles were observed in the breast CSCs when treated with 2 μM of Rott, indicating that Rott not only increased the number of vacuoles, but also increased the number of mature autophagosomes formed per cell (Figure 2b). We next counted and graded CSCs based on abundance of LC3-II positive staining. The number of LC3-II positive CSCs and severity of autophagic response per cell (number of autophagosomes present per cell) was increased following Rott treatment at 48 h (Figure 2c).

However the co-treated breast CSCs with Rott and autophagy inhibitors Baf, 3-MA or CHX inhibited autophagy (Figure 2d, e and f). 3-MA is a phosphatidylinositol 3-kinase class-III enzyme inhibitor which is essential for the autophagic process and the autophagy inducing potential of Rott was partially reverted with 3-MA, indicating that inhibition of phosphatidylinositol 3-kinase class III enzyme reduced the number of cells undergoing autophagy. Baf is a potent and specific inhibitor of vacuolar H + ATPase (V-ATPase) which stops the acidification of lysosomes during the formation of autophagosomes and slows down the lipidation of LC3 protein. CHX, a small molecule inhibitor of protein synthesis, blocks the elongation phase of eukaryotic translation.

### Molecular evidence of regulation of autophagy by rottlerin

**Figure 3:**
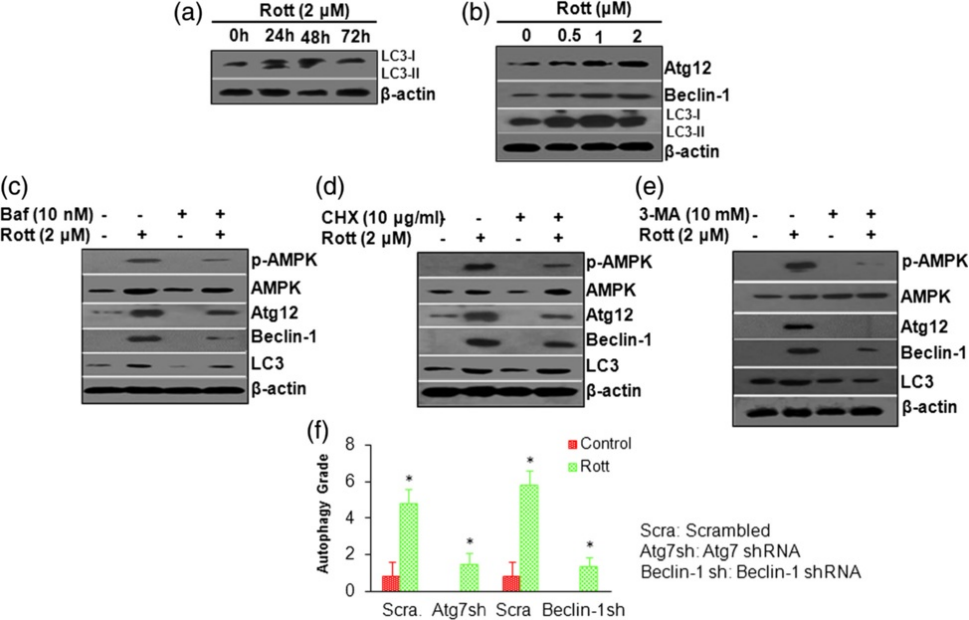
**Rottlerin induced autophagy in breast CSCs. (a)** Breast CSCs were grown in complete stem cell culture medium and treated with Rott (2 μM) for 0, 24, 48 and 72 h. Representative blots showing the concentration dependent effect of Rott on breast CSCs. Rott regulates autophagy-related genes in breast CSCs. Conversion from LC3-I to LC3-II by Rott. The Western blot analysis was performed to measure the expression of LC3. β-actin was used as a loading control. **(b)** Breast CSCs were grown in complete stem cell culture medium and treated with Rott (0, 0.5, 1 and 2 μM) for 48 h. The Western blot analysis was performed to measure the expression of Atg12, Beclin-1, LC3 and β-actin. **(c)** Breast CSCs were pre-incubated with Baf (10 nM) for 2 h, followed by treatment with Rott (2 μM) in complete stem cell culture medium for 48 h. The Western blot analysis was performed to measure the expression of p-AMPK, AMPK, Atg12, Beclin-1, LC3 and β-actin. **(d)** Breast CSCs were pre-incubated with CHX (10 μg/ml) for 2 h, followed by treatment with Rott (2 μM) for 48 h. The Western blot analysis was performed to measure the expression of p-AMPK, AMPK, Atg12, Beclin-1, LC3 and β-actin. **(e)** Breast CSCs were pre-incubated with 3-MA (10 mM) for 2 h, followed by treatment with Rott (2 μM) for 48 h. The Western blot analysis was performed to measure the expression of p-AMPK, AMPK, Atg12, Beclin-1, LC3 and β-actin. **(f)** Atg7 or Beclin-1 shRNA inhibits Rott-induced autophagy. pEGFP-LC3-positive breast CSCs were transduced with scrambled, Atg7 shRNA or Beclin-1 shRNA and treated with Rott (2 μM) for 24–48 h. Autophagy was measured as described in Figure 2. Data are reported as the mean ± standard error (SE). n = 5, *P < 0•05.

**Figure 4:**
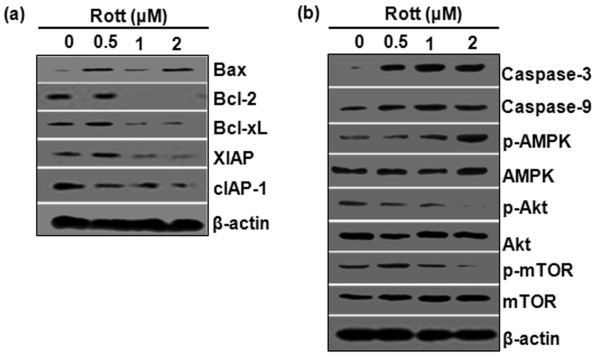
**Rottlerin induced apoptosis in breast CSCs. (a)** Breast CSCs were grown in complete stem cell culture medium and treated with Rott (0, 0.5, 1 and 2 μM) for 48 h. The Western blot analysis was performed to measure the expression of Bax, Bcl-2, Bcl-xL, XIAP, cIAP-1, and β-actin. **(b)** Breast CSCs were grown in complete stem cell culture medium and treated with Rott (0, 0.5, 1 and 2 μM) for 48 h. The Western blot analysis was performed to measure the expression of active caspase-3, active caspase-9, p-AMPK, AMPK, p-Akt, Akt, p-mTOR, mTOR and β-actin.

To determine whether Rott regulates autophagy at 24–48 h, first we examined the levels of LC3-II, which is an LC3-phosphatidylethanolamine conjugate and a promising autophagosomal marker. Rott induced an increase in the lipidated form of LC3 (LC3-II) at 24–48 h, further indicating the induction of autophagy at early stage (Figure 3a). However, Rott-induced conversion of LC3-I to LC3-II was not observed at 72 h. We next measured the expression of autophagy-related proteins, LC3, Atg12, Beclin-1 in breast CSCs treated with Rott (Figure 3b). The levels of Atg12 and Beclin-1 expression were increased in a dose dependent manner following treatment with Rott. These results indicate that Rott stimulated not only the conversion of a fraction of LC3-I into LC3-II but also caused the accumulation of Atg12 and Beclin-1 proteins. The cellular levels of Bcl-2, Bcl-xL, XIAP and cIAP-1 proteins were significantly decreased after the treatments with Rott for 48 h (Figure 4a). The accumulation of Atg12 and Beclin-1 proteins may be mediated by the reduction in Bcl-2 and Bcl-xL expression. To assess how the pro-apoptotic effect of Rott was linked to the autophagies signal, we used autophagy inhibitors (Baf and 3-MA), and protein synthesis inhibitor (CHX). Treatment of breast CSCs with Baf, 3-MA or CHX inhibited Rott-induced conversion of LC3, and induction of Atg12 and Beclin-1 (Figure 3c, d and e), suggesting that Rott has potential to induce autophagy in CSCs.

Inhibition of Atg7 or Beclin-1 by shRNA suppressed Rott-induced autophagy in breast CSCs. We have recently demonstrated the requirement of Atg7 or Beclin-1 in Rott-induced autophagy [25,26]. To investigate the mechanism of Rott-induced autophagy in breast CSCs, we inhibited autophagy by Atg7 shRNA or Beclin-1 shRNA. These plasmids have been previously validated in our laboratory [25,26]. As shown in (Figure 3f), overexpression of either Atg7 shRNA or Beclin-1 shRNA suppressed Rott-induced autophagy, suggesting the requirement of these genes in Rott-induced autophagy.

### Rottlerin-induced autophagy is mediated via activation of AMPK pathway

Several recent studies have shown that activation of AMPK is important in regulating autophagy. We wanted to test whether this was the case in our model. The western blot data showed that Rott activated AMPK by phosphorylating it at Thr-172 in breast CSCs. Further, to confirm the role of AMPK in Rott induced autophagy, we exposed the CSCs to Baf, 3-MA or CHX before treating with Rott. Treatment of breast CSCs with Baf, 3-MA, and CHX inhibited Rott-induced activation of AMPK (Figure 3c, d and e). Interestingly, blocking AMPK activation also blocked the expression of LC3, Atg12 and Beclin-1 in breast CSCs, indicating that AMPK also mediates the effect of Rott on autophagy. These results strongly establish that AMPK is a major regulator of Rott-induced autophagy in breast CSCs.

### Rottlerin induced apoptotic cell death via inhibition of Akt/mTOR pathway and activation of caspases

Akt/mTOR signaling pathway is involved in the regulation of cell cycle, cellular transformation, cell growth, and tumorigenesis. To investigate the upstream inhibition of mTOR by Rott, we examined Ser473 phosphorylation of Akt. As shown in (Figure 4b), treatment with Rott decreased the levels of phosphorylated Akt and mTOR in breast CSCs. These data suggest that Rott induces apoptosis by inhibiting Akt/mTOR pathway. To gain further insight into the mechanism by which Rott induces cell death, we examined the effects of Rott on the expression of apoptosis-related proteins (Figure 4a and b). Treatment of breast CSCs with Rott resulted in cleavage of caspase-3 and caspase-9. Furthermore, the levels of IAP family proteins, such as XIAP and cIAP-1, which bind to caspases and lead to their inactivation, were downregulated by Rott treatment (Figure 4a). Moreover, the cellular levels of anti-apoptotic Bcl-2 and Bcl-xL proteins were significantly decreased, whereas pro-apoptotic Bax level was increased in response to Rott, indicating Rott induced cell death in CSCs due to an increase in the relative ratio of Bax/Bcl-2 (and/or Bcl-xL) expression.

### Rottlerin induced apoptotic cell death in breast CSCs

**Figure 5:**
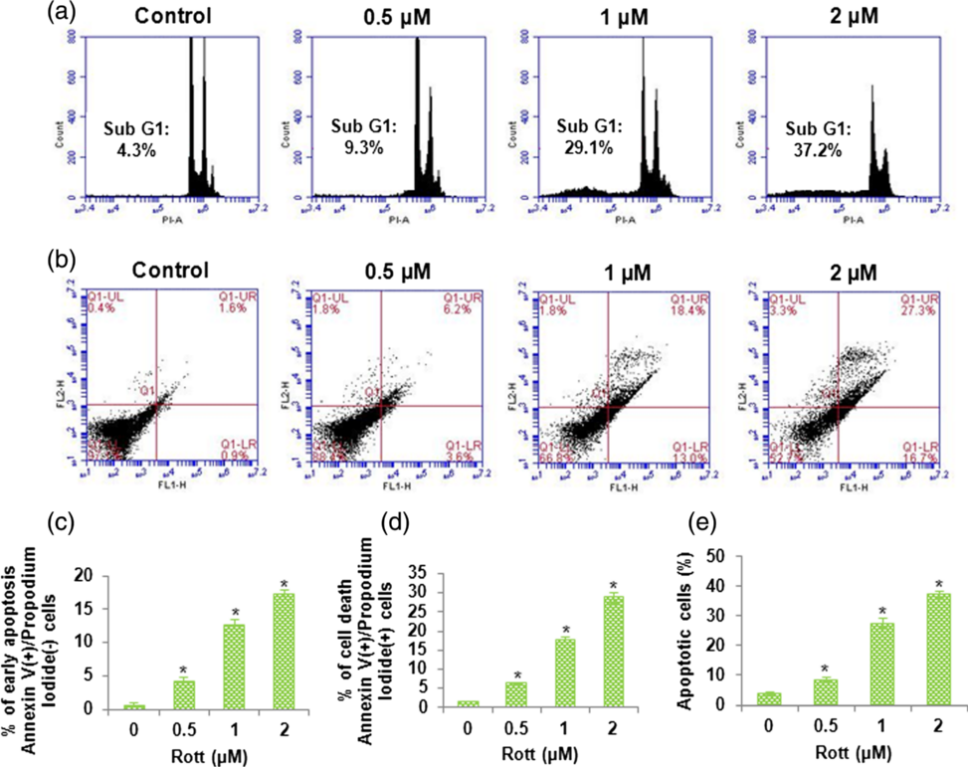
**Apoptotic cell-death measured by flow cytometer in breast CSCs. (a)** Breast CSCs were treated with Rott (0, 0.5, 1 and 2 μM) in complete stem cell culture medium for 48 h and apoptosis was measured by PI staining followed by flow cytometry. Data are the means of triplicate experiments. **(b)** Time-course evaluation of spontaneous apoptosis of breast CSCs treated with Rott (0, 0.5, 1 and 2 μM) in complete stem cell culture medium for 48 h and apoptosis was measured by annexin-V/PI staining followed by flow cytometry. Data are the means of triplicate experiments. Representative histograms are shown of Rott treated breast CSCs stained with annexin-V and propidium iodide. After 48 h of culture, three populations of cells were observed: viable cells (negatively stained, lower left quadrants); early apoptotic cells (annexin-V positive and propidium iodide negative, lower right quadrant) and cells in the late stages of apoptosis (annexin-V and propidium iodide positive, upper right quadrants). By increasing Rott concentration at 48 h, a greater number of breast CSCs underwent the early and late stages of apoptosis. **(c)** Kinetic evaluation of cells undergoing the early stage of apoptosis in breast CSCs. Cells staining with annexin-V only, showing the early stage of apoptosis (% early apoptosis) **(d)** Kinetic evaluation of cells underwent the late stage of apoptosis in breast CSCs. Cells staining with annexin-V alone and with both annexin-V and propidium iodide were combined, giving the total number of cells at both the early and late stages of apoptosis (% cell death). **(e)** Kinetic evaluation of cells underwent apoptotic cell death in breast CSCs. Data are reported as the mean ± standard error (SE) of percentage of cells. *n* = 5, *P < 0.05 when compared with Rott treated in an identical manner.

We studied the effect of Rott on the induction of autophagy leads to the apoptotic cell death in breast CSCs by using C6-flow cytometer. Rott did not significantly induce apoptosis in breast CSCs at 24 and 48 h (data not shown), but significantly induced apoptotic cell death at 72 h (Figure 5a to e). Breast CSCs treated with different concentration of Rott (0, 0.5, 1 and 2 μM) underwent apoptosis as assessed by flowcytomer using propidium iodide (PI), and annexin-V/PI staining (Figure 5a, b, c, d and e). Cells underwent apoptosis quickly showed an increase in annexin-V binding by increasing Rott concentration but excluded PI (early apoptosis). At later time-points, the percentage of PI-staining of breast CSCs gradually increased (late apoptosis). Therefore, we report here both the percentage of early apoptosis (which indicates annexin-V-positive cells only) and the percentage cell death, which indicates the total number of annexin-V-FITC-plus PI staining cells and is representative of populations containing cells at both early and late stages of apoptosis.

**Figure 6:**
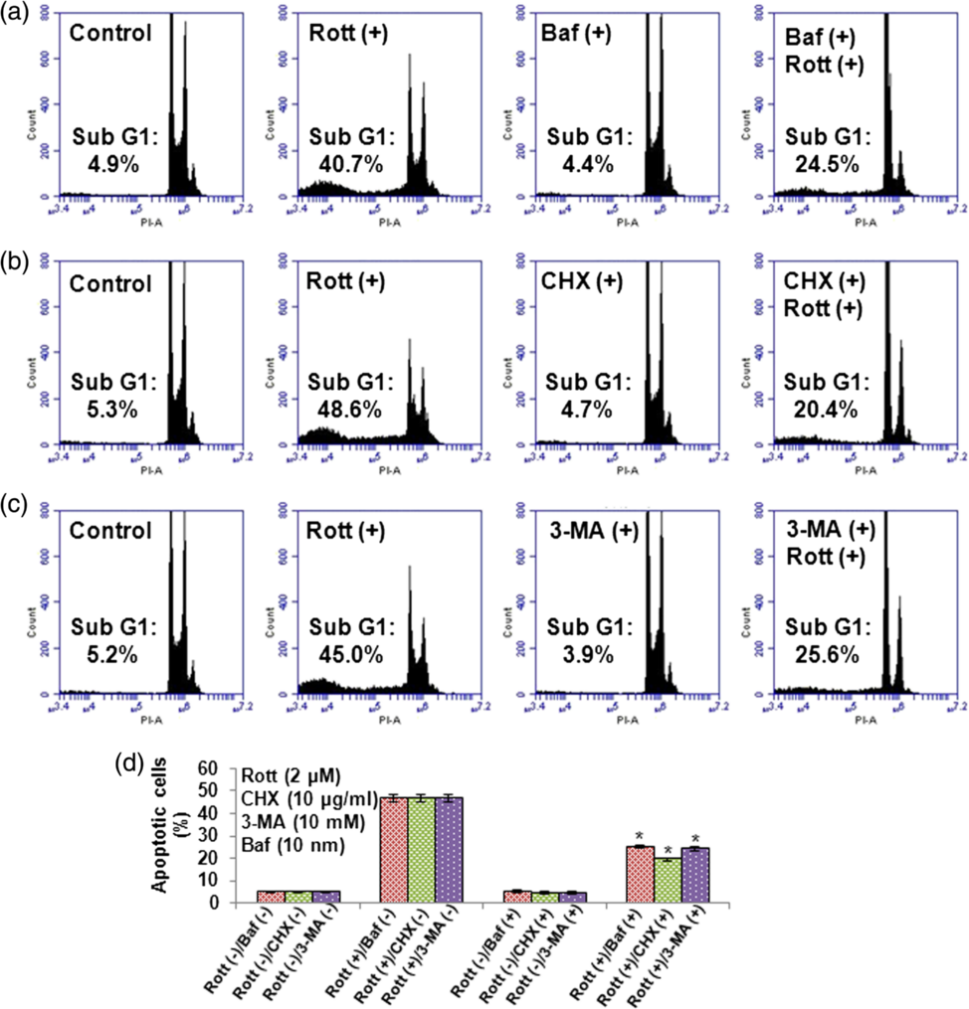
**Apoptotic cell-death measured by PI staining in breast CSCs.** Cells were co-treated with Rott (2 μM) and Baf (10 nM), 3-MA (10 mM) or CHX (10 μg/ml). **(a)** Rott (2 μM) and Baf (10 nM), **(b)** Rott (2 μM) and CHX (10 μg/ml), and **(c)** Rott (2 μM) and 3-MA (10 mM) in complete stem cell culture medium for 48 h. Apoptosis was measured by PI staining followed by flow cytometry. Data are the means of triplicate experiments. **(d)** Kinetic evaluation of cells underwent apoptotic cell death in breast CSCs. Data are reported as the mean ± standard error (SE) of percentage of cells. n = 5, *P < 0.05 when compared with Rott treated in an identical manner.

**Figure 7:**
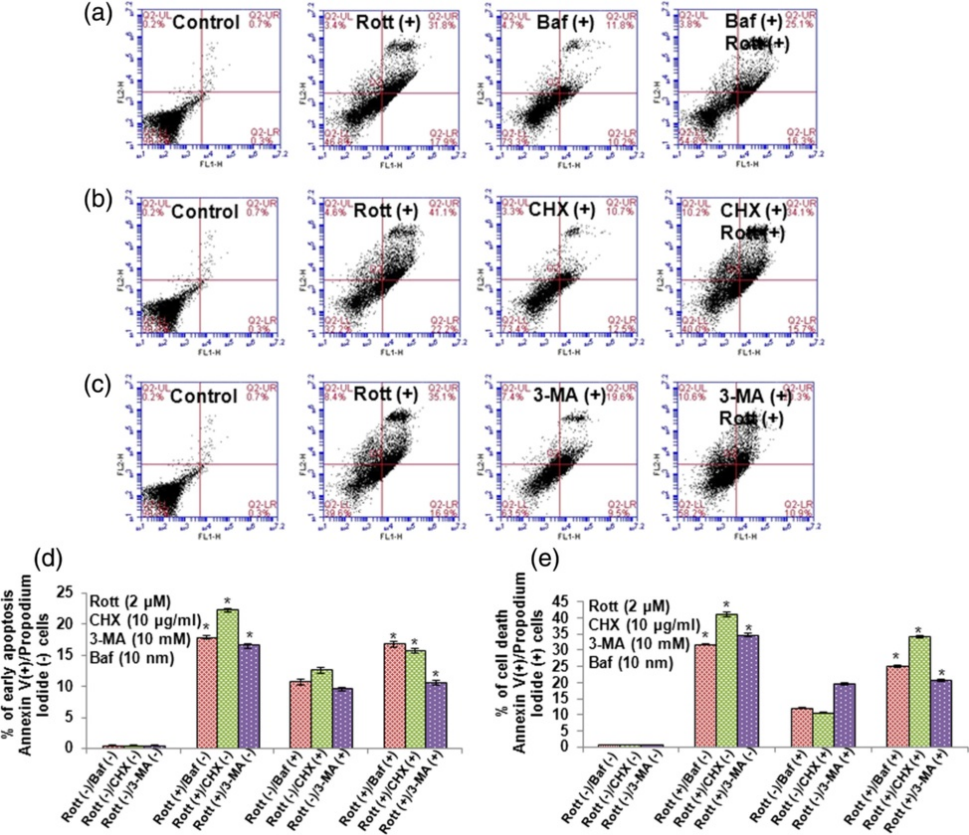
**Apoptotic cell-death measured by annexin-V/PI staining in breast CSCs.** Cells were co-treated with Rott (2 μM) and Baf (10 nM), 3-MA (10 mM) or CHX (10 μg/ml) **(a)** Rott (2 μM) and Baf (10 nM), **(b)** Rott (2 μM) and CHX (10 μg/ml), and **(c)** Rott (2 μM) and 3-MA (10 mM) in complete stem cell culture medium for 48 h. Apoptosis was measured by annexin-V/PI staining followed by flow cytometry. Data are the means of triplicate experiments. Representative histograms are shown of co-treated with **(a)** Rott (2 μM) and Baf (10 nM), **(b)** Rott (2 μM) and CHX (10 μg/ml), and **(c)** Rott (2 μM) and 3-MA (10 mM) breast CSCs stained with annexin-V and propidium iodide. After 48 h of culture, three populations of cells were observed: viable cells (negatively stained, lower left quadrants); early apoptotic cells (annexin-V positive and propidium iodide negative, lower right quadrant) and cells in the late stages of apoptosis (annexin-V and propidium iodide positive, upper right quadrants). By increasing Rott concentration at 48 h, a greater number of breast CSCs underwent the early and late stages of apoptosis. **(d)** Kinetic evaluation of cells undergoing the early stage of apoptosis in breast CSCs. Cells staining with annexin-V only, showing the early stage of apoptosis (% early apoptosis) **(e)** Kinetic evaluation of cells undergoing the early stage of apoptosis in breast CSCs. Cells staining with annexin-V alone and with both annexin-V and propidium iodide were combined, giving the total number of cells at both the early and late stages of apoptosis (% cell death). Data are reported as the mean ± standard error (SE) of percentage of cells. n = 5, *P < 0.05 when compared with Rott treated in an identical manner.

Further, to confirm the role of Baf, 3-MA or CHX in apoptotic cell death, we exposed breast CSCs to Baf, 3-MA or CHX before treating with Rott. Rott-induced apoptosis in breast CSCs, as assessed by PI (Figure 6a, b, c and d) and annexin-V/PI (Figure 7a, b, c, d and e) staining through flow cytometry. Pretreatment of CSCs with Baf, 3-MA or CHX inhibited Rott induced apoptosis. These data suggest that inhibition of autophagosome and protein synthesis can block Rott-induced apoptosis.

### Molecular docking between C2-domain of protein kinase C-delta and rottlerin

**Figure 8:**
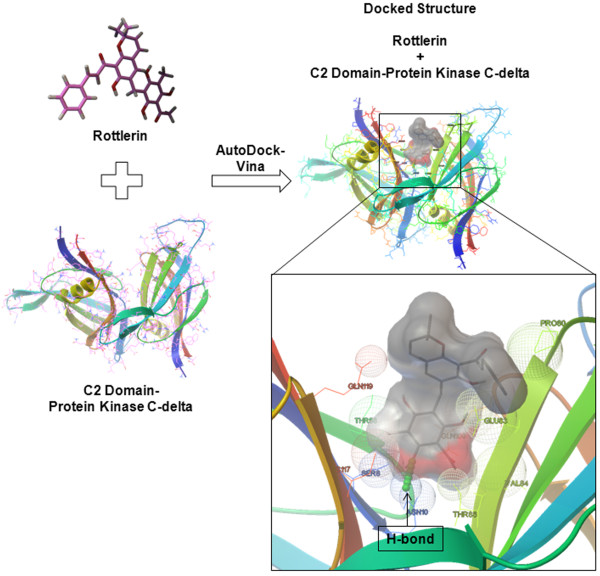
**Molecular docking between C2-domain of protein kinase C-delta and rottlerin.** Binding conformations of top ranked docked poses of Rott into C2 domain of protein kinase C-delta. Binding activity of docked structure predicted by AutoDock-Vina is only showing important residues are displayed in CPK style. The inhibitors, and part of the amino acid residues in the background were visualized in New Ribbon style using the AutoDockTools4.

Molecular docking is a computational method that attempts to predict noncovalent binding between macromolecule (receptor/protein) and a small molecule (ligand). In order to understand the induction of autophagy in CSCs in exposure to Rott we have performed molecular docking between protein kinase C-delta inhibitor (Rott) [46] and C2-domain of protein kinase C-delta [49]. We have used AutoDock-Vina [50] docking program to see the interaction between protein and ligand. There are several other docking programs (e.g. GOLD, FleX, FRED) which can be used to predict the binding affinity between protein and ligand. AutoDock-Vina showed best for carrying out blind docking between protein and ligand among them [51]. Each docking result generated top ten best binding conformations of the ligand and the best binding poses (Figure 8). The 3D view of protein–ligand interactions of the best poses generated by ADT are shown in Figure 8. As clearly showed in Figure 8, an important interactions can be found between ligand and the residues SER8, ASN10, THR58, GLU83, PRO80, VAL84, THR85, GLN109, CYS117 and GLN119 which directly participate in the catalytic mechanism of this protein. The protein-ligand complex is stabilized mainly by hydrogen bonds and hydrophobic interactions. All the top docked poses generated by each docking routine exhibited well-established bonds with one or more amino acids in the binding pocket of C2-domain of protein kinase C-delta. The top-ranked pose with lowest docked binding affinities and high docking scores is generally used as a standard selection in most of the docking programs. The best poses of C2-domain of protein kinase C-delta-Rott were generated by AutoDock-Vina. The binding affinity for Rott was found to be −7.5 Kcal/mol. The orientation and hydrogen bonding, ionic interactions of Rott with C2-domain of protein kinase C-delta active site are shown in Figure 8. These docking experiments suggest that Rott can directly bind to protein kinase C-delta.

## Discussion

In this study we demonstrated that Rott induces early autophagy as a survival strategy against late apoptosis through AMPK and Akt/mTOR cascade dependent pathways in human breast CSCs. One of the most surprising events in the early stage apoptosis by Rott treatment was the cytoplasmic vacuolation. These vacuoles were formed by Rott-induced autophagy and were identified by electron microscopy, acidic vesicular organelle staining, and transfection of green fluorescent protein-LC3. Interestingly, Rott-treated cells did not undergo cell death at 24 h, while at late time points (72 h) showed significant cell death. Rott induced autophagy at 24–48 h, as evident by formation of autophagosomes and conversion of LC3-I to LC3-II form. Rott was found to cause typical autophagy characteristics, including formation of autophagosomes and acidic vacuoles, and redistribution of LC3 at 24–48 h. These results indicate that treatment with Rott may induce autophagy at an early stage in breast CSCs. Our study for the first time demonstrates that Rott treatment induces autophagy in breast CSCs by activating AMPK pathway.

Autophagy is a catabolic process during which damaged organelles and proteins are engulfed and degraded to provide metabolic needs. Autophagy is activated in response to various kinds of stress. It is a conserved dynamic process in which intracellular membrane structures sequester proteins and organelles, which are finally delivered to lysosomes for bulk degradation and ATP generation to maintain basal cellular bioenergetics [17]. Whereas the above situations envision autophagy as a survival mechanism, autophagy can also lead to cell death under some circumstances [52]. Rott-induced apoptotic cell death was mediated through a decrease of mitochondrial membrane potential and translocation of AIF into nucleus at a late time point. Moreover, the inhibition of Rott-induced autophagy with Baf, 3-MA or CHX slows down apoptotic cell death. The most novel mechanistic aspect of this study is, perhaps, that Rott-induced autophagy may act as a survival factor against caspase-independent cell death. Treatment with Rott induced a dose- and time-dependent growth inhibition and also triggered cell death with cytoplasmic vacuolation in breast CSCs, which is consistent with the reported biological events caused by Rott in breast tumor and malignant glioma cells [53,54]. On the other hand, Rott treatment in the presence of Baf, 3-MA or CHX lead to decreased expression of LC3 when compared to the cells treated either with Rott or inhibitors alone, suggesting increased autophagic potentials. All three Baf, 3-MA, and CHX inhibit the fusion between autophagosomes and lysosomes, thus prevent the execution step of autophagy. Nonetheless, our results from flowcytometry demonstrated that autophagy inhibitors (Baf and 3-MA), and protein synthesis inhibitor (CHX) inhibits Rott-induced autophagy. Our observations are in agreement with several studies demonstrating the role of LC3 in autophagy [33,55,56].

In this study, Rott was found to induce autophagy in breast CSCs, including formation of autophagosomes, redistribution of LC3 and induction of autophagy related proteins including Atg12 and Beclin-1 at 24–48 h. The antiapoptotic protein, Bcl-2, inhibits the Beclin-1 dependent autophagy [57,58]. Rott significantly inhibited Bcl-2 and Bcl-xL expression, and induced Atg12 and Beclin-1. Furthermore, Baf, 3-MA or CHX inhibited Rott-induced conversion of LC3-I to LC3-II, and expression of autophagy-related proteins Atg12 and Beclin-1 at 24–48 h. Activation of autophagy by Rott in our model was confirmed by enhanced expression of LC3. Our results also showed that autophagy induction was associated with an increase in the expression of Beclin-1 and Atg12. Autophagy marker LC3 is a protein that is selectively incorporated into autophagosome by directly binding to LC3 and hence aggregate during autophagy. Atg12 is instrumental in the autophagic vesicle biogenesis [19]. These results indicate that Rott induces autophagy at an early stage in breast CSCs. Beclin-1 was originally discovered as a Bcl-2 interacting protein and was one of the first human proteins shown to be indispensable for autophagy [59]. Another autophagic gene Atg7 is responsible for autophagosome biogenesis [60]. Both genes are monoallelically deleted in 50–75% of cases of human sporadic breast, ovarian and prostate cancers [60]. Our study demonstrates that co-treatment of the CSCs with Rott and Baf, 3-MA or CHX inhibited the Rott-induced autophagy and slows down the apoptotic process. Therefore, Rott-induced autophagy may play some role in apoptotic cell death. Apoptosis is an important tumor suppressor mechanism that is blocked in the majority of human cancers, due to the over activation of the AMPK and Akt/mTOR pathway [47]. Activation of AMPK and Akt/mTOR pathway regulates transcription factors which modulate distinct sets of genes involved in cell cycle, apoptosis, oxidative stress and DNA repair [47]. Treatment of CSCs with Rott increased the levels of phosphorylated AMPK. Furthermore, downregulation of constitutively active Akt/mTOR and upregulation of AMPK rendered breast CSCs sensitive to Rott. Rott induced significant apoptosis in breast CSCs at 48 h by inhibiting phosphorylation of Akt and mTOR, and expression of Bcl-2, Bcl-xL, cIAP1 and XIAP, up-regulation of AMPK and Bax, and activation of caspase-3 and −9. Our results indicate that Rott causes early autophagy and late apoptosis through inhibition of Akt/mTOR pathway in human breast CSCs.

The recent study also suggests that autophagy at early stage may act as a survival mechanism against late apoptosis. Thus, inhibition of autophagy by the potent drugs or genetic means (e.g. inhibiting the expression of Atg7 and Beclin-1) may enhance the apoptosis-inducing potential of Rott in highly therapy-resistant human breast CSCs. Our study established that autophagy induced by Rott treatment was mediated by activation of AMPK pathway. Chemical inhibitors such as Baf, 3-MA or CHX not only blocked the induction of LC3, but also inhibited Rott induced expression of Atg12 in breast CSCs. Rott treatment raises cytosolic calcium levels which activate the various kinases including AMPK which is known to regulate autophagy. AMPK is also an energy sensor and is activated when there is increase in AMP/ ATP ratio, which is usually the scenario during cellular stress, the same reason for which autophagy is activated [33]. In agreement with these facts, Rott treatment activated AMPK in breast CSCs. Baf, 3-MA or CHX not only suppressed Rott induced phosphorylation of AMPK but also attenuated the expression of LC3 and Atg12. All these observations are in agreement with several studies showing that activation of AMPK leads to autophagy [33,45,61].

There are several previous studies has been confirmed that Rott acts as a very effective protein kinase C-delta inhibitor and it plays an essential role in the induction of autophagy and apoptotic cell death [15,46]. Apart from studying the effect of Rott on CSCs and the induction of autophagy and apoptotic cell death, we have also studied the computational docking between Rott and C2-domain of protein kinase C-delta. The docking results between Rott and C2-domain of protein kinase C-delta generated by AutoDock-Vina showed the strong molecular interactions between Rott and C2-domain of protein kinase C-delta. It forms hydrogen bonds, hydrophobic and ionic interactions with the important residues of the binding pocket of C2-domain of protein kinase C-delta thus stabilizing the structure of target receptor.

## Conclusions

In the present study, for the first time we conclude that Rott, a natural compound derived from the plant kamala tree (*Mallotus philippinensis*) with cytotoxic effect, could induce extensive cytoplasmic vacuolization in breast CSCs. The fact that autophagy inhibitors suppress the formation of cytoplasmic vacuolization confirmed that there might be interaction between autophagy and apoptosis induced by Rott. Therefore, Rott could affect the functions of a variety of proteins and act as a multi-target compound. The potency of Rott to induce autophagy and apoptosis at the same dose might be based on its capability to activate several pathways such as AMPK and proteasome inhibition. Further studies are needed to elucidate the complicated signal cascades induced by Rott.

## Methods

### Cell culture, reagents and antibodies

Human breast CSCs [CD44(+)/CD24(−/low)] were isolated from primary tumors and grown in M171 medium (Invitrogen, Carlsbad, CA) containing mammary epithelial growth supplement (MEGS) (Invitrogen, Carlsbad, CA) and 1% antibiotic-antimycotic (Invitrogen), and maintained in a humidified incubator with 5% CO_2_, and 37°C temperature. Rott, 3-MA, CHX, Baf, puromycin were obtained from Sigma-Aldrich Corp. (St. Louis, MO). Anti-human LC3, Atg7, Atg12, Beclin-1, Bax, Bcl-2, Bcl-XL, cIAP-1, Akt, pAkt, mTOR, p-mTOR and XIAP, AMPK, pAMPK and β-actin antibodies were obtained from Cell Signaling Technology (Danvers, MA).

### pEGFP-LC3 transfection in breast CSCs

Breast CSCs were transfected with pEGFP-LC3 plasmid using neon electroporator at 1400 V, 2-pulses for 20 ms. 30 μg of DNA was mixed with the cell suspension and electroporated by using 100 μl neon tips. After electroporation, pEGFP-LC3 transfected breast CSCs were seeded in 60 mm culture dish. After 2 days, transfected cells were selected by 10 μM neomycin, and visualized under Leica 6000B microscope with 10X objectives.

### Lentiviral particle production and Atg7 and Beclin-1 transduction

Atg7 shRNA and Beclin-1 shRNA were obtained from Open Biosystems (Lafayette, CO). Lentivirus particles were produced by triple transfection of HEK 293 T cells. Packaging 293 T cells were plated in 10 cm plates at a cell density of 5 × 10^6^. Transfection of packaging cells and infection of breast CSCs were carried out using standard protocols with some modifications. In brief, 293 T cells were transfected with 8 mg of plasmid and 4 mg of lentiviral vector using lipid transfection (Lipofectamine-2000) according to the manufacturer’s protocol. Viral supernatants were collected and concentrated by adding PEG-it virus precipitation solution (SBI System Biosciences, Mountain View, CA). Breast CSCs were transduced with viral particles expressing scrambled, Atg7 shRNA or Beclin-1 shRNA.

### Vacuolated cell enumeration

Cells were seeded in 6-well plates at a density of 1 × 10^4^ cells/well in complete stem cell culture medium and incubated overnight. Cells were then treated with various concentration of Rott (0, 0.5, 1 and 2 μM) for 48 h. Vacuolated cells were counted using fluorescent microscope in at least 100 cells for each condition.

### Immunofluorescence assay

Cells were grown on fibronectin-coated coverslips (Beckton Dickinson, Bedford, MA), and treated with Rott (0, 0.5, 1 and 2 μM), washed in PBS, and fixed for 15 min in 4% paraformaldehyde. Cells were permeabilized in 0.1% Triton X-100, washed and blocked in 10% normal goat serum. After blocking, cells were incubated with primary antibody (1:100) for overnight at 4°C, washed with PBS and incubated with fluorescently labeled secondary antibody (1:200) along with 4, 6-diamido-2-phenylindole hydrochloride (DAPI) (1 mg/ml) for 1 h at room temperature. Finally, coverslips were washed and mounted using vectashield (Vector Laboratories, Burlington, CA). Isotype-specific negative controls were included with each staining. Stained cells were mounted and visualized under Leica 6000B microscope with 100X objectives. The number of cells expressing punctate and the number of punctate per cell were counted manually.

### Nuclear staining with DAPI

After Rott treatment, adherent cells were fixed for 20 min at room temperature with 4% paraformaldehyde and permeablized for 10 min with 0.2% Triton X-100 in PBS. After PBS washes, cells were stained with 4, 6-diamido-2-phenylindole hydrochloride (DAPI) in PBS at the concentration of 1 mg/ml for 15 min at room temperature. Cells were then washed with PBS and visualized using Leica 6000B microscope with 100X objectives.

### XTT based cell viability assay

Breast CSCs (1 × 10^4^ in 200 μl culture medium per well) were seeded in 96-well plate (flat bottom), treated with Rott (0, 0.5, 1 and 2 μM), and incubated for 48 h at 37°C with 5% CO_2_. Before the end of the experiment, 50 μl XTT labeling mixture (final concentration, 125 μM XTT (sodium 2,3-Bis(2-methoxy-4-nitro-5-sulfophenyl)-2H-tetrazolium-5-carboxanilide inner salt) and 25 μM PMS (phenazine methosulphate) per well was added and plates were incubated for further 4 h at 37°C and 5% CO_2_. The spectrophotometric absorbance of the sample was measured using a microtitre plate (ELISA) reader. The wavelength to measure absorbance of the formazon product was 450 nm, and the reference wavelength was 650 nm.

### Measurement of apoptotic cell death by flow cytometer

Breast CSCs (10000 cells/well) were seeded in 6 well plate and exposed to Rott (0, 0.5, 1 and 2 μM). Cells were then washed in PBS and collected by trypsinization, and fixed overnight in 70% glacial ethanol. Next day cells were washed in PBS and resuspended in 1 mL of PBS containing 50 μg/mL RNase and incubated at 37°C for 2 h. 50 μg/mL propidium iodide (PI) added in resuspended cells and then incubated for 60 min in dark at 4°C. Cell cycle analysis was performed by flow cytometry (Becton Dickinson, Franklin Lakes, NJ, USA), and the population of cells in each phase was calculated using the Cell Quest software program. Each experiment was conducted three times.

Breast CSCs (10000 cells/well) were seeded in 6-well plates and exposed to Rott (0, 0.5, 1 and 2 μM). Treated cells were washed twice with cold PBS and resuspended in buffer at a concentration of 10^6^ cells per ml. Cells were mixed with 10 μl of fluoresceine isothiocyanate (FITC)-conjugated annexin-V reagent and 10 μl of 3 mM propidium iodide (PI). After 15 min incubation at room temperature in the dark and further washings, samples were analyzed by flow cytometry. Flow cytometry was performed with a FACScan analyzer (Becton Dickinson, Franklin Lakes, NJ, USA) with15 mW argon ion laser (488 nm) and Cell Quest software. Annexin-V staining was detected in the FL1 channel, whereas PI staining was monitored in the FL2 channel: appropriate quadrants were set and the percentage of cells negative for stains (viable cells), positive for annexin-V (apoptotic cells) and positive for PI (dead cells) were acquired.

### Electron microscopy

To demonstrate the induction of autophagy in Rott-treated breast CSCs, cells were treated with (0, 0.5, 1 and 2 μM) of Rott for 24 h; cells were harvested by trypsinization, washed and fixed in 2% glutaraldehyde in 0.1 M phosphate buffer, then post-fixed in 1% osmium tetroxide buffer. After dehydration in a graded series of ethanol, cells were embedded in spur resin. Thin sections (60 nm) were cut on an Ultramicrotome. The sectioned grids were stained with saturated solutions of uranyl acetate and lead citrate. The sections were examined by electron microscope.

### Preparation of whole-cell lysates

After treatment with Rott (0, 0.5, 1 and 2 μM), breast CSCs were pelleted by centrifugation at 1,000 X rpm for 5 min and washed once with PBS. Cells were then resuspended in RIPA buffer (50 mM Tris–HCl, pH 7.5, 150 mM NaCl, 1% v/v Nonidet P-40, 0.5% v/v sodium deoxycholate and 0.1% SDS) supplemented with protease inhibitor cocktail (Sigma) and phosphatase inhibitor cocktail (Sigma), and lysed on ice by sonicating for 5 s and 5–10 pulses. The lysates were centrifuged for 20 min at 12,000 X g and supernatant was collected and used for further experiments.

### Western blot analysis

Total cellular lysates were obtained by lysing cells in a buffer containing RIPA buffer and a mixture of protease and phosphatase inhibitors. Lysates were sonicated for 5 s and 5–10 pulses, centrifuged for 20 min at 12,000 X g and stored at -80°C. Equal amounts of lysate proteins (50–60 μg total protein) were electrophoretically separated by 10% sodium dodecyl sulfate-polyacrylamide gel electrophoresis (SDS-PAGE) and transferred to nitrocellulose membrane. Nitrocellulose blots were blocked with 5% nonfat dry milk in TBS buffer (20 mM Tris–HCl, pH 7.4, and 500 mM NaCl), and incubated with primary antibody in TBS-T (TBS and 0.01% Tween-20) overnight at 4°C. Immunoblots were washed three times (5, 5 and 5 min each) with TBS-T followed by 1–2 h incubation with secondary antibody. Chemiluminescence reactions were carried out with the Super Signal West Pico substrate (Thermo Fisher, Waltham, MA).

### AutoDock-Vina docking file preparation and running docking program

Ligands (Rott) were designed by using ACD/ChemSketch Freeware software (http://www.acdlabs.com/resources/freeware/chems-ketch/). Open Babel (An open chemical toolbox) software used to convert 3D structure of ligand (.MOL file) into.PDB file. Ligands were optimized by using Graphical User Interface program AutoDockTools4 (ADT) [62]. Protein (C2-domain of protein kinase C-delta) was downloaded from RCSB-Protein Data Bank (PDB-ID: 1BDY) [49] and edited in.txt file. Protein was optimized by using ADT [62]. Intermediary steps, such as.pdbqt files for protein and ligands preparation and grid box creation were completed using ADT. ADT assigned charges, solvation parameters and fragmental volumes to the protein. AutoDock saved the prepared file in pdbqt format. AutoGrid was used for the preparation of the grid map using a grid box. The grid size was set to 58 × 56 × 74 xyz points with grid spacing of 0.375 Å and grid center was designated at dimensions (x, y, and z): -3.085, 28.517 and 37.651. AutoDock-Vina was employed for docking using protein and ligand information along with grid box properties in the configuration file. AutoDock-Vina employs iterated local search global optimizer [50]. During the docking procedure, both the protein and ligands are considered as rigid. The results less than 1.0 Å in positional root-mean-square deviation (RMSD) was clustered together and represented by the result with the most favorable free energy of binding. The pose with lowest energy of binding or binding affinity was extracted and aligned with receptor structure for further analysis.

## Abbreviations

3-MA: 3-Methyladenine; AMPK: Adenosine monophosphate-activated protein kinase; Baf: Bafilomycin; CSCs: Cancer stem cells; CHX: Cycloheximide; Rott: Rottlerin; XTT: (sodium 2,3-Bis(2-methoxy-4-nitro-5-sulfophenyl)-2H-tetrazolium-5-carboxanilide inner salt).

## Competing interests

The authors declare no competing interests.

## Authors’ contributions

DK conceived the idea, performed the experiments and analyzed the data; SS and RK designed the experiments; DK wrote the manuscript. All authors read and approved the manuscript.
